# Antidepressant Potential of Quercetin and its *Glycoside* Derivatives: A Comprehensive Review and Update

**DOI:** 10.3389/fphar.2022.865376

**Published:** 2022-04-08

**Authors:** Shen Chen, Yueheng Tang, Yang Gao, Kexin Nie, Hongzhan Wang, Hao Su, Zhi Wang, Fuer Lu, Wenya Huang, Hui Dong

**Affiliations:** ^1^ Institute of Integrated Traditional Chinese and Western Medicine, Tongji Hospital, Tongji Medical College, Huazhong University of Science and Technology, Wuhan, China; ^2^ Grade 2017 of Integrated Traditional Chinese and Western Clinical Medicine, Second Clinical School, Tongji Hospital, Tongji Medical College, Huazhong University of Science and Technology, Wuhan, China; ^3^ Department of Integrated Traditional Chinese and Western Medicine, Tongji Hospital, Tongji Medical College, Huazhong University of Science and Technology, Wuhan, China

**Keywords:** quercetin, antidepressant, glycoside derivatives, isoquercetin, hyperin, rutin, quercetin 4′-O-glucoside, avicularin

## Abstract

Depression is a global health problem with growing prevalence rates and serious impacts on the daily life of patients. However, the side effects of currently used antidepressants greatly reduce the compliance of patients. Quercetin is a flavonol present in fruits, vegetables, and Traditional Chinese medicine (TCM) that has been proved to have various pharmacological effects such as anti-depressant, anti-cancer, antibacterial, antioxidant, anti-inflammatory, and neuroprotective. This review summarizes the evidence for the pharmacological application of quercetin to treat depression. We clarified the mechanisms of quercetin regulating the levels of neurotransmitters, promoting the regeneration of hippocampal neurons, improving hypothalamic-pituitary-adrenal (HPA) axis dysfunction, and reducing inflammatory states and anti-oxidative stress. We also summarized the antidepressant effects of some quercetin glycoside derivatives to provide a reference for further research and clinical application.

## 1 Introduction

Depression is a common mental disorder composed of emotional, neurovegetative, and cognitive symptoms that seriously diminishes the quality of life of patients and worsens the personal ability to work and learn (Lecrubier, 2001; Joo, 2017; Lee et al., 2017; Malhi and Mann, 2018). Clinically, depression has different manifestations due to its various combinations of symptoms, leading to diagnostic difficulties, while the most typical symptoms are persistent sadness and a lack of interest or pleasure in previously rewarding or enjoyable activities. According to the World Health Organization (WHO), approximately 280 million people worldwide suffered from depression in 2021, with a prevalence of about 3.8%. Depression can also lead to suicide at its worst and over 700,000 people die due to suicide every year (World Health Organization, 2020). Hence, depression can seriously threaten everyone’s health and quality of life.

Previous studies have verified that the occurrence of depression is related to multiple biological factors. The pathogenesis of depression includes nerve damage and aplastic disorder (Liu et al., 2017), *hypothalamic-pituitary-adrenal* (HPA) axis dysfunction (Aubry, 2013), monoamine nervous system hypofunction (Yohn et al., 2017; Delva and Stanwood, 2021), inflammation (Felger, 2019), oxidative stress (Bhatt et al., 2020), and genetic and psychosocial factors (Ménard et al., 2016). Due to the complex pathogenesis of depression and its serious harm to patients, early identification and intervention for patients with depression are of great importance. Currently, selective serotonin reuptake inhibitors (SSRIs), serotonin and norepinephrine reuptake inhibitors (SNRIs), tricyclic and tetracyclic antidepressants, and monoamine oxidase inhibitors (MAOIs) are clinically used to treat depression. (Berton and Nestler, 2006). These drugs target only one aspect of the pathogenesis, which makes most of them slow-acting. Simultaneously, the compliance of depression patients is greatly reduced due to side effects such as dizziness, vomiting, and libido reduction. Some drugs can also raise blood glucose levels and body weight, increasing the risk of other diseases (Carvalho et al., 2016). Furthermore, 15–30% of patients can present drug resistance during treatment (Lucas et al., 2017). Therefore, it is urgent to search for safe and effective antidepressants that act on multiple targets and pathways.

Traditional Chinese Medicine (TCM) is a holistic system based on empirical therapies such as acupuncture and herbal medicine (Xu et al., 2013), with significant advantages regarding syndrome differentiation, holistic treatment, and comprehensive treatment through multi-component, multi-target, and multi-pathway mechanisms. Recently, increasing studies have confirmed that many TCM have antidepressant effects (Yang et al., 2018; Wang et al., 2019; Li et al., 2020a; Feng et al., 2022). For example, icariin can exert antidepressant-like effects by promoting the antioxidant status and reducing inflammation (Liu et al., 2015). Berberine and ginsenosides can improve depression-like behaviors in rats by regulating the levels of plasma corticosterone (CORT) and adrenocorticotropic hormone (ACTH) (Zhang et al., 2021). XiaoYaoSan, a famous Chinese herbal formula, significantly ameliorates chronic unpredictable mild stress (CUMS)-induced depression-like behaviors in rats by reversing metabolic perturbations (Liu et al., 2019).

**FIGURE 1:**
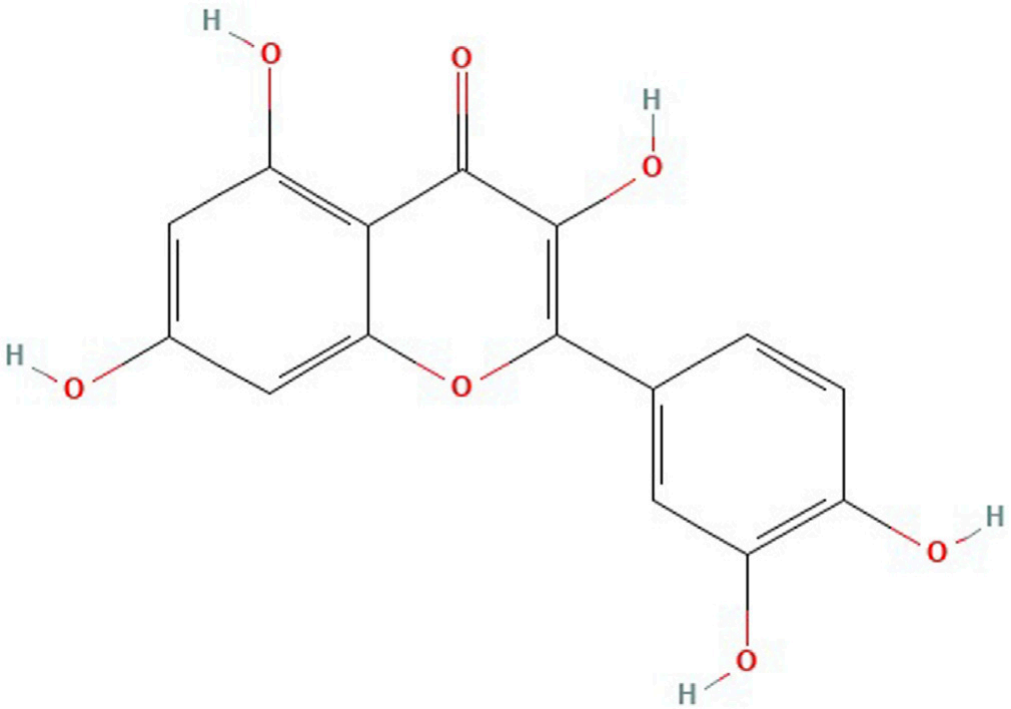
Chemical structure of quercetin.

Quercetin (C5H10O7; Figure 1), a flavonol widely present in nature in the form of glucosides (Ulusoy and Sanlier, 2020), is one of the main components of different TCM, including Radix Bupleuri, Mulberry leaves, Sophorae Fructus, Inulae Flos, and Crataegi Fructus (Ding et al., 2021a; Shi et al., 2021; Tu et al., 2022). Many studies have demonstrated that quercetin has a wide range of pharmacological effects such as anti-cancer, antibacterial, anti-oxidation, and memory impairment improvement (Horowitz and Zunszain, 2015; Suganthy et al., 2016; Babaei et al., 2018a; Kashyap et al., 2019). Recently, an increasing number of studies have focused on the treatment of depression with quercetin and its glycoside derivatives.

Therefore, in this review, we searched for studies on the antidepressant effects of quercetin and its glycoside derivatives in PubMed, Web of Science, ScienceDirect, Chinese National Knowledge Infrastructure (CNKI), and Wanfang Data Resource System Chinese Science databases from January 2000 to February 2022. Search terms included “quercetin,” “quercetin glycoside derivatives,” “isoquercetin,” “hyperin,” “rutin,” “*quercetin 4′-O-glucoside*,” “*quercetin 3-O-α-* *l* *-arabinofuranoside*,” “depression,” “neuroplasticity,” “inflammation,” “cell regeneration,” “oxidative stress.” The inclusion criteria were as follows: 1) explored the antidepressant effects of quercetin or its glycoside derivatives; 2) animals with depression-like behavior after modeling were selected for *in vivo* experiments, and neurons or microglia for *in vitro* experiments; 3) behavioral tests, oxidative stress indicators, inflammatory factors, and neurons and microglia viability were used for depression assessment. Finally, we reviewed 55 studies on the antidepressant effects of quercetin and its glycoside derivatives and their mechanisms to provide a reference for future research and clinical applications.

## 2 Experimental Evidence of the Antidepressant Effects of Quercetin *In vivo* and *In vitro*

The studies on the antidepressant effects of quercetin were mainly based on rats and mice models with different stress stimuli. The most common evaluation methods are behavioral tests such as forced swimming test (FST), tail suspension test (TST), sucrose preference test (SPT), open field test (OFT), light and dark box test (LDA), and elevated plus maze (EPM). Most of these studies focused on *in vivo* experiments, and there were only a few *in vitro*. Table 1 presents relevant information of *in vivo* studies and the mechanisms explored in the *in vitro* studies will be described below. Overall, they demonstrated that quercetin has high antidepressant activity. However, there is still a need for more in-depth studies regarding its antidepressant effects *in vitro*.

**TABLE 1:** The *in vivo* studies on the antidepressant effects of quercetin.

Animal	Experimental model	Modeling method	Usage	Dosage	Evaluation Method	Function	Refs
Mice	Balb/c mice	Aflatoxin B1 intervention	p.o	30 mg/kg	FST, OFT, MWM, NOR	Quercetin reduced the immobility time in FST, increased the time spent in left in OFT, reduced the anxiety-like behaviors in EPM and MWM and increased the exploration time in NOR	Gugliandolo et al. (2020)
	C57BL/6N mice	CSDS	Dietary intervention	0.5, 2 g/kg	TST, SPT, OFT, EPM, social interaction test	Quercetin reduced the immobility time in TST and increased the time spent both in the left zone in OFT and in the open arm in EPM.	Zhang et al. (2020b)
	C57BL/6J mice	Estrogen receptor α missing	p.o	100 mg/kg	TST, FST	Quercetin reduced the immobility time of mice both in TST and in FST.	Wang et al. (2021b)
	C57BL/6J mice	LPS intervention	i.p	30, 60 mg/kg	TST, FST, neurons and microglia activities	Quercetin reduced the immobility time both in TST and in FST, suppressed the activation of microglia and alleviated the loss of DA neurons	Han et al. (2021)
	ICR mice	LPS intervention	i.p	10 mg/kg	TST, FST, SPT, OFT	Quercetin reduced the immobility time in both TST and FST, reversing the anhedonia-like activities in SPT	Sun et al. (2021)
	KM mice	CUMS	p.o	10, 20, 40 mg/kg	FST, SPT, OFT	Quercetin reduced the immobility time in FST and enhanced sucrose preference in SPT	Guan et al. (2021b)
	NMRI mice	mTBI	p.o	50 mg/kg	OPT, EPM, zero maze, light-dark box, HPA axis activity	Quercetin reduced anxiety-like behaviors in behavioral tests, decreasing the levels of adrenocorticotropic hormones and corticosterone	Kosari-Nasab et al. (2019)
	Swiss mice	OB	p.o	25 mg/kg	TST, FST, OFT, splash test	Quercetin reduced the immobility time in both TST and FST, reducing locomotor activities in OFT.	Holzmann et al. (2015)
	Swiss mice	GBH intervention	p.o	30 mg/kg	FST, SPT, OFT, EPM	Quercetin demonstrated a partial improvement in the number of entries in EPM and reduced the immobility time in FST.	Bicca et al. (2021)
	Swiss albino mice	CRF antagonist intervention	p.o	10, 20, 40 mg/kg	FST, social interaction test, locomotor activity	Quercetin reduced the immobility time in FST and increased their social interaction time in social interaction test	Bhutada et al. (2010)
	Swiss albino mice	CUMS	p.o	25 mg/kg	TST, MFST, OFT	Quercetin reduced the immobility time of mice both in TST and in MFST, increasing field crossings and the time spent in left in OFT	Khan et al. (2019)
	Swiss albino mice	CUS	p.o	30 mg/kg	MWM, NOR	Quercetin increased the number of platform crossings and the time spent in searching platform in the target quadrant in MWM, increasing discrimination between novel and familiar objects in NOR.	Mehta et al. (2017a)
	Swiss albino mice	CUS	p.o	30 mg/kg	OFT, SPT, EPM, passive avoidance step-through task	Quercetin enhanced sucrose preference and exploration behaviors of mice	Mehta et al. (2017b)
	Swiss albino mice	72 h of sleep deprivation	p.o	25, 50 mg/kg	TST, OFT, NOR	Quercetin reduced the immobility time of mice in TST, increased number of lines crossed in OFT and increased the duration of exploration of the novel object in NOR.	Eduviere et al. (2021)
	Wistar albino mice	Immobilization stress	i.p	20 mg/kg	FST, LDA, EPM, MWM, antioxidant enzyme activity	Quercetin reduced the immobility time in FST, improved the time spent in open arms in EPM, increased the time spent in the light box in LDA, decreased the time to reach the hidden platform in MWM and reduced the levels of MDA in their brains	Samad et al. (2018)
Pigs	Guinea pigs	Lumateperone intervention	p.o	50 mg/kg	FST, OFT	Quercetin reduced the immobility time in FST, causing a significant increase in crossing squares in OFT.	El-Haroun et al. (2021)
Rats	SD rats	CUMS	p.o	10, 50 mg/kg	SPT	Quercetin reduced the depressive behaviors of animals in SPT.	Guan et al. (2021a)
	SD rats	LPS intervention	p.o	40 mg/kg	FST, SPT, OFT, MWM, Y maze	Quercetin reduced the immobility time in FST, improving sugar water preference index in SPT and new arm preference index in OFT.	Fang et al. (2019)
	SD rats (pregnant)	MS	Dietary intervention	0.03%	FST, OFT, EPM	Quercetin reduced the immobility time in FST and increased the number of entries in the open arms in OFT.	Donoso et al. (2020)
	Wistar rats	ADR intervention	i.p	60 mg/kg	FST, OFT, EPM	Quercetin reduced the immobility time in FST and the anxiety-like behaviors in both OFT and EPM.	Merzoug et al. (2014)
	Wistar rats	Alloxan induces diabetes	p.o	100 mg/kg	FST, OFT, EPM, social interaction test	Quercetin increased the time spent in swimming and struggling in FST, increased the time spent in left and the total distance traveled in OFT, improved the time spent in open arms and the locomotive distance in EPM and enhanced the sociability in social interaction test	Toumi et al. (2019)
	Wistar rats	CUMS	p.o	50 mg/kg	FST, SPT	Quercetin reduced the immobility time in FST, increased the sucrose consumption in SPT and inhibited the iNOS and MDA in animals	Bin-Jaliah, (2021)
	Wistar rats	CUMS	p.o	25, 50 mg/kg	FST, OFT, fluid consumption test	Quercetin reduced the immobility time in FST, augmenting the number of line crossings, total distance traveled, the number of entries in left zone and time in the left zone in OFT.	Quraishi et al. (2018)
	Wistar rats	DMH induces colorectal cancer	p.o	50 mg/kg	FST, OFT	Quercetin reduced the immobility time of mice in FST, increasing walking and feeding frequency in OFT.	Sadighparvar et al. (2020)
	Wistar rats	OB	p.o	20, 40 mg/kg	FST, OFT	Quercetin reduced ambulation, rearing, defecation and increased the grooming/licking episodes in OFT, reducing the characteristic hyperactivity and the immobility time in FST.	Rinwa and Kumar, (2013)
	Wistar rats	Rotenone intervention	p.o	50 mg/kg	SPT, OFT, NOR, MWM, beam walking test, inclined plane test, footprint test, social interaction test	Quercetin increased the intake of sugar in SPT, reduced the latency to move and increased the number of squares crossed in OFT.	Madiha et al. (2021)
	Wistar albino rats	CUMS	i.p	30 mg/kg	FST, SPT, locomotor activity	Quercetin reduced the immobility time in FST, improving sucrose preference in SPT.	Şahin et al. (2020)
	Wistar albino rats (pregnant)	NaF intervention	p.o	20 mg/kg	OFT, maze learning test, levels of monoamines	Quercetin increased head elevation, hind limb elevation, sniffing, grooming, auditory startle, pivoting scores of animals in OFT, restoring the levels of Ach in the cerebral cortex of developing rat brain	Mesram et al. (2017)
Zebrafish	*Danio rerio* zebrafish	LPS intervention	i.p	50, 100 mg/kg	Novel tank diving test, light–dark chamber test, inflammation examination and antioxidant enzyme activity	Quercetin increased the time spent in the top zone and the number of entries in the top zone in novel tank diving test, increasing the time spent in the light zone and the number of entries in the light zone in the light–dark chamber test	Singh et al. (2022)
	*Danio rerio* zebrafish	Bisphenol A intervention	Directly spiked into water	2.96 μM	Novel tank diving test, light/dark preference test	Quercetin ameliorated the BPA-induced alteration in time spent in top zone, the number of transitions to top zone and latency to enter top zone in novel tank diving test	Sahoo et al. (2020)
	*Danio rerio* zebrafish	Aluminum chloride intervention	Directly spiked into water	2 μl	Novel tank test, light/dark preference test, native area test	Quercetin increased the time spent in the top zone in novel tank test	Mani et al. (2018)
	Wild-type zebrafish	—	Directly spiked into water	0.01, 0.1, 1, 10, 100, 1,000 μg/L	Novel tank test, shoaling behavior test, anxiety behavior test	Quercetin increased the shoaling tendency and the latency to enter the upper half in shoaling behavior test, increasing the total time in the upper and the total number of midline transitions in the anxiety behavior test	Zhang et al. (2020a)

SPT, sucrose preference test; FST, forced swimming test; MFST, modified forced swimming test; TST, tail suspension test; OFT, open field test; LDA, light and dark box test; EPM, elevated plus maze; NOR, novel object recognition test; NMRI, Naval Medical Research Institute; CUMS, chronic unpredictable mild stress; CUS, chronic unpredictable stress; MS, maternal separation procedure; CSDS, chronic social defeated stress; OB, olfactory bulbectomy; TNF, tumor necrosis factor; IL, Interleukin; SOD, superoxide dismutase; CAT, catalase; GSH, glutathione; MDA, malondialdehyde; GPx, glutathione peroxidase; MAO, Monoamine oxidase; 5-HT, serotonin; BDNF, Brain-derived neurotrophic factor; HPA, hypothalamic-pituitary-adrenal; ACTH, adrenocorticotropic hormone; CORT, corticosterone; Ach, acetylcholine; AchE, acetylcholinesterase; LPS, Lipopolysaccharide; DMH, Dimethyl hydrazine; mTBI, Mild Traumatic Brain Injury; ADR, adriamycin; GBH, glyphosate-based herbicide; CRF, corticotrophin releasing factor; TrkB, Tyrosine Kinase receptor B; AKT, decreased protein kinase B; ERK1/2, extracellular regulated protein kinases; NaF, Sodium fluoride; KM, Kunming; ICR, Institute of Cancer Research; SD, Sprague Dawley.

## 3 Clinical Evidence of the Antidepressant Effects of Quercetin

At present, few clinical trials have applied quercetin as a single drug to explore its pharmacological effects. Additionally, no relevant clinical trials have evaluated its antidepressant effects. Thus, we retrieved clinical studies using Chinese herbal formulas as interventions in which quercetin was the main component (Sun et al., 2018; Li et al., 2019; Wei et al., 2021). For example, the effects of Chaihu Shugansan in improving depression when used as monotherapy were significantly better than antidepressants such as fluoxetine, paroxetine, and duloxetine, and were comparable to these antidepressants in enhancing recovery rate (Wang et al., 2012). Danzhi Xiaoyaosan has similar clinical comprehensive effects to antidepressants (Wang et al., 2021b). Therefore, quercetin can work synergistically with other components to improve depression. Meanwhile, clinical trials evaluating the antidepressant effects of quercetin as a monotherapy are still required.

## 4 Mechanisms Underlying the Antidepressant Effects of Quercetin

### 4.1 Regulation of Neurotransmitter Levels

The monoaminergic hypothesis states that the dysfunction of the monoaminergic neurotransmitter system in the body can result in depression (Govindarajulu et al., 2021). Catecholamines, composed of dopamine (DA), norepinephrine (NE), and epinephrine, are monoamine neurotransmitters, similar to indoleamines, especially serotonin (5-HT). MAOIs were the first drugs applied to alleviate the symptoms of depression. First, as a treatment for tuberculosis, MAOIs have been confirmed to improve patients’ mood, inhibit the oxidation of monoamines, and increase the concentration of monoamine neurotransmitters in the extracellular fluid of the brain (Yohn et al., 2017). Subsequent clinical observations and experiments demonstrated the lower levels of monoamines and metabolites in the cerebrospinal fluid of depression patients (Ogawa et al., 2018), and that the depletion of 5-HT and NE with reserpine can lead to depression (Blackburn, 2019), providing evidence for the monoamine hypothesis. Recently, increasing studies have attempted to update this hypothesis. The prevailing view combines it with neuroplasticity for the shortcomings, based on the finding that 5-HT can modulate neuroplasticity-related signaling pathways in fully mature brains (Mahar et al., 2014; Kraus et al., 2017). Meanwhile, several studies have summarized the 5-HT, glutamate (Glu), and γ-aminobutyric acid (GABA) systems into the “monoamine-Glu/GABA long neural circuit” hypothesis. In this case, the rebalancing of GABA interneurons and Glu pyramidal neurons is considered the rate-limiting step in depression treatments and would explain the latency of antidepressants (Li, 2020). Although these hypotheses are constantly updated, the high correlation between monoamine neurotransmitters and the onset of depression has always been well recognized.

Furthermore, in the brains of mice and rats, the levels of 5-HT were higher after quercetin treatment (Khan et al., 2019; Madiha et al., 2021). Although Samad et al. did not find changes in 5-HT concentrations after quercetin intervention, the proliferation of plasma 5-HIAA (a 5-HT metabolite) induced by stress was reversed in their study (Samad et al., 2018). Except directly increasing the levels of 5-HT and reducing the levels of its products, studies have also found that quercetin can inhibit the metabolism of 5-HT by monoamine oxidase (MAO), thereby preventing the occurrence of low 5-HT concentrations in the body (Bandaruk et al., 2012; Herraiz and Guillén, 2018).

The theory based on NE and its receptors was first proposed by Schildkraut et al. (1965). This theory proposes that inadequate contents of NE in the central nervous system of the brain can lead to depression (Maletic et al., 2017). Recently, quercetin has been proved to restore reduced NE levels in rats exposed to sodium fluoride (NaF) (Mesram et al., 2017). Although the effects of quercetin on NE receptors in the brain remain unclear, some indications might be extracted from an *in vitro* study which found that quercetin-3-O-glucuronide (Q3G), a metabolite of quercetin in humans, could inhibit α2 and β2-adrenergic receptors expressed by NE and human breast cancer cells and protect cells under stress by reducing free radicals (Yamazaki et al., 2014). Altogether, these experiments evidenced that quercetin could exert antidepressant effects by regulating the function of the monoaminergic nervous system.

Besides monoaminergic neurotransmitters, quercetin can improve depression-like behaviors by regulating choline, amino acid neurotransmitters, and their corresponding receptors. Moreover, quercetin has a good binding affinity for GABAα receptors and attenuates depressive symptoms by antagonizing somatostatin to stimulate GABAergic interneurons (Hossain et al., 2021). Previous studies have also confirmed that quercetin can reduce the levels of acetylcholinesterase (AchE) in animals (Samad et al., 2018) and the hyperactivity of cholinergic nerves, leading to hypoadrenergic function, which would cause depression (Teneralli et al., 2021). Therefore, the inhibitory effect of quercetin on cholinergic nerves is one of its mechanisms that can relieve depressive symptoms.

### 4.2 Promotion of Cell Regeneration in the Hippocampus

The regeneration hypothesis comprehends another view of depression pathogenesis and is supported by increasing evidence. For example, X-ray-irradiated mice present depressive-like behaviors due to neurogenesis disorders that can not be improved by antidepressants (Santarelli et al., 2003). Additionally, antidepressants can promote neurogenesis in the dentate gyrus of the hippocampus (Eliwa et al., 2017), indicating that neurogenesis is necessary for antidepressants to function.

At present, many studies have demonstrated that quercetin promotes cell regeneration in the hippocampus. For example, Karimipour et al. (2019) described the impact of quercetin on neurogenesis using 5-Bromodeoxyuridinc (BrdU) to label neural stem cells. They found that quercetin promoted the proliferation and differentiation of neural stem cells in adult rats with Alzheimer’s disease. Moreover, Baral et al. (2017) found that the number of dentate gyrus neurons rose and the proliferation accelerated after oral administration of quercetin in mice. Interestingly, quercetin and Q3G had opposite regulatory effects in this study: the viability of human embryonic neuronal cells was reduced after quercetin exposure, followed by decreased phosphorylation levels of protein kinase B (AKT). Meanwhile, Q3G promoted cell proliferation and AKT phosphorylation, suggesting that quercetin might have bidirectional regulatory capacities.

Besides the fact that quercetin could directly increase the number of hippocampal neurons and glial cells, the regulation of brain-derived neurotrophic factor (BDNF) by quercetin can indirectly promote neurogenesis. BDNF is a neurotrophic factor that binds to its high-affinity receptor tyrosine kinase receptor B (TrkB) to promote the survival, proliferation, and maturation of adult olfactory bulb and dentate gyrus neural progenitor cells (Eliwa et al., 2017). Moreover, BDNF has been validated as a key factor that promotes synaptic plasticity to exert antidepressant effects (Erickson et al., 2012; Zhang et al., 2016). Quercetin can alleviate depressive-like behaviors induced by lipopolysaccharide (LPS) in rats by increasing the levels of BDNF and TrkB in the hippocampus and prefrontal cortex (Fang et al., 2019). Meanwhile, the ability of quercetin to upregulate BDNF levels has been validated in MS model rats, polychlorinated biphenyl-treated rats, and 1,2-dimethylhydrazine-induced colorectal cancer-complicated depression rats (Selvakumar et al., 2018; Donoso et al., 2020; Sadighparvar et al., 2020). Overall, the activation of BDNF/TrkB-related signaling pathways by quercetin reinforces its role in the promotion of cell regeneration.

### 4.3 Improvement of Neuroplasticity

#### 4.3.1 Improvement of Hypothalamic-Pituitary-Adrenal Axis Dysfunction

The HPA axis is an important part of the body’s neuroendocrine system. The corticotropin-releasing hormone (CRH) stimulates the anterior pituitary to release ACTH in response to stress, then ACTH stimulates the adrenal glands to secrete glucocorticoids (GC) (Sheng et al., 2020). Meanwhile, in the internal environment, GC promotes negative regulation feedback on the hypothalamus and pituitary by inhibiting CRH and ACTH production (Gjerstad et al., 2018). It is generally assumed that high GC levels induced by chronic stress or dysregulation of GC negative feedback receptors contribute to hippocampal and hypothalamic damage, finally leading to neurogenic impairment (Podgorny and Gulyaeva, 2021). Moreover, GC can participate in the reduction of hippocampal and hypothalamic blood flow in dogs (Yamazaki et al., 2021). The excess of ACTH can also cause damage to the hippocampus and hypothalamus by enhancing GC secretion. Additionally, CORT is a major GC component, and the excess of CORT leads to decreased expression of total AKT and total glucose transporters-4 (GLUT4), thereby affecting glucose uptake by brain cells (Calvo-Ochoa and Arias, 2015). This dysregulation of the HPA axis caused by the injured brain leads to the proliferation of oligodendrocytes, exacerbating depressive symptoms (Herman et al., 2016; Tertil et al., 2018). In summary, the dysregulation of the HPA axis is closely related to depression.

Quercetin can also exert antidepressant effects by inhibiting the hyperactivity of the HPA axis, which has been confirmed by multiple studies. For example, Donoso et al. (2020) demonstrated that quercetin improved depressive symptoms in maternally isolated model mice, as assessed by EPM, OFT, and FST, with the reduction of CORT levels. Similarly, Merzoug et al. (2014) reported that quercetin could alleviate the doxorubicin-induced anxiety-like depression behaviors in mice, and, after adrenaline intervention, the elevated CORT levels also reduced. Moreover, Kosari-Nasab et al. found that quercetin was as effective as diazepam in reducing ACTH and CORT levels in mice with mild traumatic brain injury (Kosari-Nasab et al., 2019). Nevertheless, with the continuous emergence of drugs targeting the HPA axis (Ding et al., 2021b), a positive control group based on mifepristone or arginine vasopressin V1BR antagonists, for example, can be used to verify the antidepressant efficacy of quercetin in the future (Mikulska et al., 2021).

Regarding the mechanisms, current studies have focused on inhibiting the expression of corticotropin-releasing factor messenger ribonucleic acid (CRF mRNA) (Bhutada et al., 2010; Kawabata et al., 2010), controlling the levels of interleukin (IL)-1β to prevent continued activation of the HPA axis (Mkhize et al., 2017), and promoting GABAergic neurons to regulate the termination of the HPA axis in response to stress (Hossain et al., 2021). These mechanisms can not be simply integrated and the precise mechanisms by which quercetin inhibits the hyperactivity of the HPA axis are not fully understood, so further studies are required. It is worth mentioning that when Demir et al. (2016) used rats with diabetes to understand these mechanisms, they found that quercetin did not affect ACTH, total CORT, and free CORT concentrations, showing that the antidepressant effects of quercetin were independent of the HPA axis (Mehta et al., 2017b).

#### 4.3.2 Reduction of Inflammation

The blood-brain barrier is the first defense of the brain against external aggression. In the central nervous system, non-specific immunity removes debris from necrotic nerve cells centered on microglia. However, increasing studies have demonstrated that cytokines secreted by microglia can trigger the development of various neuropsychiatric disorders, including depression (Barnes et al., 2017; Ng et al., 2018; Fakra and Marotte, 2021; Rengasamy et al., 2021). Patients undergoing immunotherapy with cytokines are frequently depressed, which will be improved after receiving ibuprofen, a non-steroidal anti-inflammatory drug (NSAID) with antidepressant-like effects (Fang et al., 2019). The antidepressants currently used in clinical practice can also alleviate inflammatory responses in the brain (Guan et al., 2021c). Animal experiments have shown that rats in LPS-induced inflammatory states exhibited depression-like behaviors and microglia activation (Fang et al., 2019). Meanwhile, long-term activation of microglia with continuous release of cytokines and chemokines result in a chronic inflammatory state, finally leading to neuronal cell damage through multiple mechanisms (Ge et al., 2015; Felger et al., 2016; Huang et al., 2020). Altogether, these results supported that the inflammatory states of the body are closely linked to depression pathogenesis.

Furthermore, the anti-inflammatory effects of quercetin have been thoroughly confirmed *in vivo* (Britti et al., 2017; Ou et al., 2020; Sato and Mukai, 2020). Many studies have shown that the anti-inflammatory effects of quercetin might reduce neuropsychiatric symptoms. Lee et al. (2020) have demonstrated that quercetin can significantly reduce IL-6 and IL-1β levels in LPS-treated rats, thereby improving anxiety-like symptoms. These results were also verified in CUMS-treated mice (Mehta et al., 2017b). The levels of nuclear factor (erythroid-2 related) factor 2 (Nrf2), an anti-inflammatory-related transcription factor in the hippocampus of mice, can be upregulated by quercetin, reducing depression-like behaviors (Guan et al., 2021c). Moreover, since astrocyte reactivation occurs when microglia cause inflammation (Liddelow et al., 2017; Li et al., 2020b), some studies have measured microglia-induced inflammatory states by astrocyte reactivation. Additionally, the ingestion of food containing a certain amount of quercetin alleviates depressive behaviors in CUMS-model mice, due to lower reactivation of astrocytes in the prefrontal cortex and hippocampus of these mice than those on a normal diet (Zhang et al., 2020b).

Finally, further studies concentrating on microglia and astrocytes are required to elucidate the reduction of inflammation in the hippocampus. Different *in vitro* studies have indicated that quercetin can inhibit pro-inflammatory mediators and attenuate the proliferation of microglia (Wu et al., 2020; Han et al., 2021; Luo et al., 2021). Quercetin can also promote the expression of astrocyte-specific molecules such as glial fibrillary acidic protein (GFAP), glutamine synthetase (GS), and ceruloplasmin (CP), thereby inhibiting the production of pro-inflammatory cytokines and chemokines (Sharma et al., 2007).

#### 4.3.3 Anti-Oxidation

Oxidative stress is another important factor that impairs neuroplasticity, which in turn can lead to depression. The antioxidant system in the body can be divided into two categories: enzyme antioxidant systems, including superoxide dismutase (SOD), catalase (CAT), and glutathione peroxidase (GSH-Px) (He et al., 2017); and non-enzymatic antioxidant systems such as ergothioneine, glutathione, melatonin, *α*-lipoic acid, as well as various vitamins and trace elements (Mirończuk-Chodakowska et al., 2018). Under normal conditions, the antioxidant system provides a shield to neurons against the adverse effects of reactive oxygen and nitrogen free radicals. Once the level of oxidative free radicals exceeds the detoxification ability of the antioxidant system, it will directly cause neuronal damage or increased levels of pro-inflammatory cytokines, finally resulting in depression-like behaviors (Mehta et al., 2017b).

Quercetin has been shown to have excellent anti-oxidative stress properties (Godoy et al., 2017), the most intuitive performance is reducing the levels of hydrogen peroxide (H_2_O_2_). For example, Selvakumar et al. (2012) observed that H_2_O_2_ levels in the hippocampus of rats exposed to polychlorinated biphenyls (PCBs) reduced after quercetin administration*.* Since MAO produces H_2_O_2_ by catalyzing the oxidative deamination that consumes monoamine neurotransmitters, it can also be used to measure H_2_O_2_ formation *in vivo* (Laban and Saadabadi, 2021)*.* Several studies have demonstrated that quercetin inhibits MAO. Therefore, quercetin can be applied as an MAO inhibitor (Bandaruk et al., 2014; Dhiman et al., 2019)*. Overall,* these results suggested that quercetin could protect neurons from oxidative stress by inhibiting H_2_O_2_ formation.

Moreover, quercetin can scavenge free radicals by regulating both enzymatic and non-enzymatic antioxidant systems. In CUMS-model mice, quercetin can reduce oxidative stress markers, including hydrogen peroxide and thiobarbituric acid reactive substances (TBARS), and simultaneously increase SOD and CAT activities (Mehta et al., 2017b). Previously, Şahin et al. (2020) have reduced the content of malondialdehyde in the brain of CUMS-model rats *via* intraperitoneal injection of quercetin. Along with elevated SOD and CAT activities, they observed the growth of glutathione in the brain, indicating that quercetin might regulate not only enzymatic but also non-enzymatic antioxidant systems. Additionally, they found that quercetin could protect model rats against vas deferens dysfunction, suggesting that quercetin has the potential to reduce depressive complications (Şahin et al., 2020). Another study has shown that quercetin had a significant effect on 24-h paradoxical sleep deprivation (PSD)-induced mice, as GSH levels were elevated in the prefrontal cortex, hippocampus, and striatum after quercetin treatment (Kanazawa et al., 2016). Guan et al. (2021a) found that the serum levels of iron, copper, and calcium increased, while magnesium, zinc, selenium, and cobalt levels significantly reduced in CUMS rats, and quercetin restored the levels of these elements, demonstrating that it could regulate trace elements in the brain. Moreover, the beneficial effects of quercetin could also be observed in other animal models, such as guinea pigs and zebrafish (El-Haroun et al., 2021; Singh et al., 2022). These studies confirmed that quercetin exerted antidepressant effects by regulating the activity of enzymatic and non-enzymatic antioxidant systems.

#### 4.3.4 Inhibition of the N-Methyl-d-aspartate Receptor and Synthesis of Nitric Oxide

The subclass of ionotropic glutamate receptors N-Methyl-d-aspartate Receptor (NMDAR) mainly mediates neuroplasticity pathways to promote learning and memory formation (Franchini et al., 2020; Stroebel and Paoletti, 2021). The overstimulation of NMDAR increases calcium influxes in neurons, which activates neuronal nitric oxide synthase (NOS) and raises Nitric Oxide (NO) concentrations, ultimately leading to depression development (Adell, 2020; Kang et al., 2020).

Holzmann et al. (2015) have demonstrated that quercetin alleviates depressive-like behaviors in olfactory bulbectomy mice, which was reversed by pretreatment with NMDA, indicating that the inhibition of the NMDAR contributed to the antidepressant effects of quercetin. Quercetin can also inhibit NO synthesis. Current studies found a significant elevation in NOS and NO levels in animal models of depressive-like behaviors, while quercetin treatment alleviated these behaviors and reversed NOS and NO abnormalities (Mehta et al., 2017b; Bin-Jaliah, 2021; Guan et al., 2021c). Therefore, the antidepressant effects of quercetin depend on the inhibition of NMDAR activity, NOS expression, and NO production (Jakaria et al., 2019a; Anggreini et al., 2019; Islam et al., 2021).

## 5 Research on the Antidepressant Effects of Quercetin Glycoside Derivatives and Related Mechanisms

**FIGURE 2:**
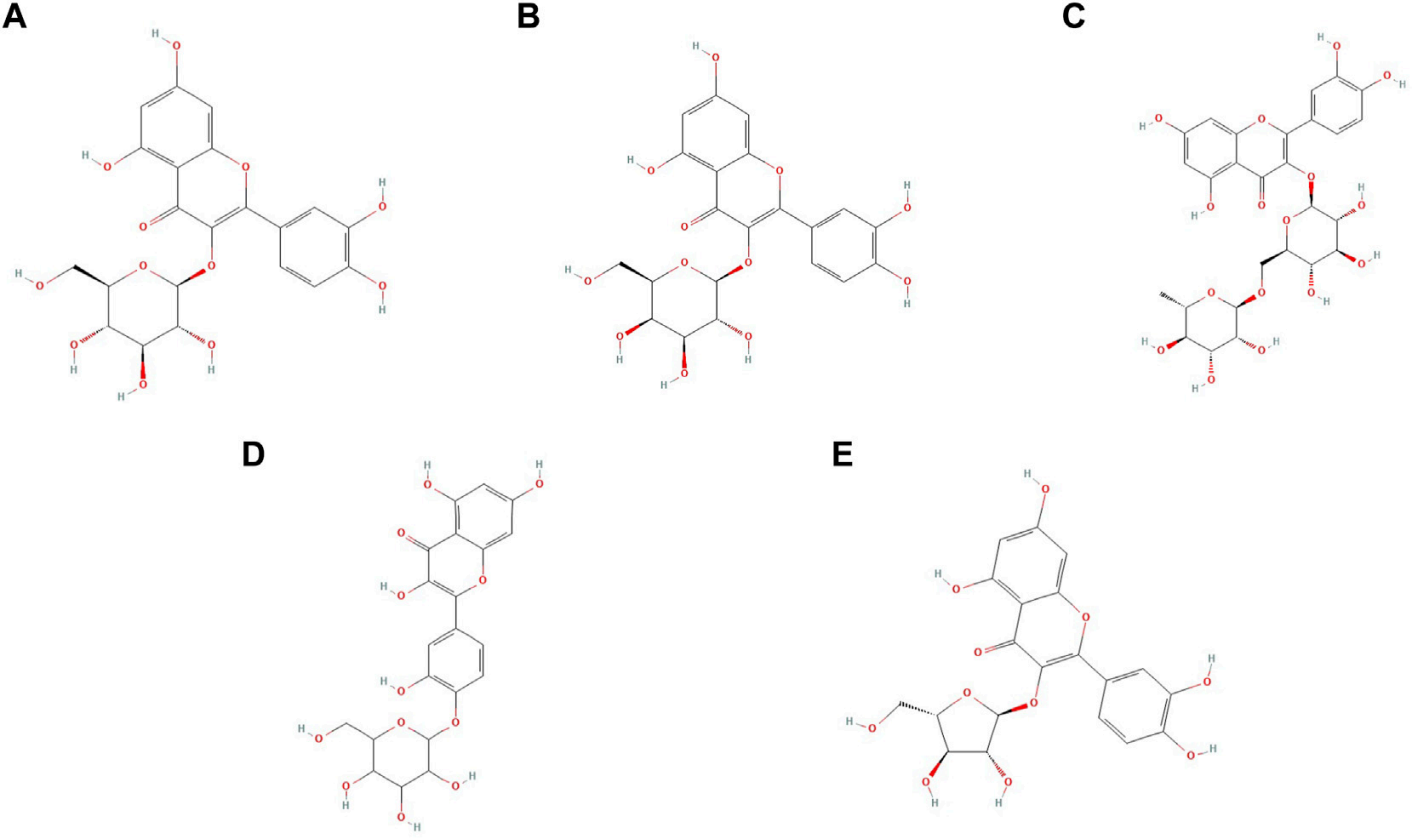
Chemical structures of quercetin glycoside derivatives: **(A)** isoquercetin; **(B)** hyperin; **(C)** rutin; **(D)** quercetin; 4′-O-glucoside; **(E)** quercetin-3-α-l-arabinofuranoside.

Quercetin mainly exists in the form of glucosides in nature. Quercetin glucosides can not pass through the cell membrane due to their weak lipid solubility (Shao et al., 2019). Hence, enzymatic conversion is commonly used to produce quercetin monomers and improve bioavailability (Manzoor et al., 2021). Additionally, the conversion of glucosides into aglycones can also be used, resulting in quercetin glycoside derivatives. Quercetin glycoside derivatives can be synthesized by glycosylation at 3-OH with monosaccharides such as glucose, galactose, rhamnose, and xylose (Magar and Sohng, 2020). Figure 2 shows the chemical structures of some quercetin glycoside derivatives with antidepressant activity, and Table 2 presents the in vivo studies on the antidepressant effects of quercetin glycoside derivatives.

**TABLE 2:** The *in vivo* studies on the antidepressant effects of quercetin glycoside derivatives.

Derivatives	Animal	Experimental model	Modeling method	Usage	Dosage	Evaluation Method	Function	Refs
Isoquercetin	Rats	CD rats	—	p.o	0.6 mg/kg	FST, locomotor activity	Isoquercetin reduced the immobility time in FST.	Butterweck et al. (2004)
The mixture of 47% quercetin 4′-O-rhamnoside and 53% isoquercetin	Mice	ICR mice	—	p.o	0.01, 0.1, 0.5, 1, 2 mg/kg	FST, OFT	The mixture reduced the immobility time in FST and decreased the events of total crossings and rearings in OFT.	Martínez-Hernández et al. (2021)
Hyperin	Mice	CF1 mice	—	i.p	10, 20 mg/kg	FST, OFT, locomotor activity	Hyperin reduced the immobility time of mice in FST, exploratory behaviors in OFT and motor activity	Haas et al. (2011)
		ICR mice	—	p.o	10, 20, 30 mg/kg	TST, FST, OFT	Hyperin reduced the immobility time in both TST and FST.	Zheng et al. (2012)
		Swiss albino mice	—	p.o	10, 20, 40 mg/kg	TST, FST, OFT, spontaneous locomotor activity	Hyperin reduced the immobility in the FST and TST, without affecting locomotor activity of mice	Orzelska-Górka et al. (2019)
	Rats	Wistar rats	—	i.p	1.8 mg/kg	FST	Hyperin reduced the immobility time in FST.	Haas et al. (2011)
	Zebra fish	*Danio rerio* mice	Exposure to 3% ethanol	Directly spiked into water	3%, 10%	Novel tank test	Hyperin increased the time spent in the top zone in novel tank test	Hadi and Troy (2014)
Rutin	Mice	Swiss mice	CUS	p.o	30 mg/kg	TST, FST, OFT, NOR	Rutin reduced the immobility time and behavioral despair in both TST and FST.	Yusha’u et al. (2017)
		Swiss mice	CUS	p.o	100 mg/kg	OFT, SPT, EPM	Rutin reduced the immobility time and protected the CUS-induced hippocampal neuronal loss	Parashar et al. (2017)
		NMRI mice (first day of pregnancy)	MS	p.o	10, 50, 100 mg/kg	FST	Rutin reduced the immobility time in FST.	Anjomshoa et al. (2020)
	Rats	Wistar rats	CUMS	p.o	50 mg/kg	FST, OFT, fluid consumption test	Rutin augmented the number of line crossings, total distance traveled, the number of entries and the time spent in the left zone in OFT, decreasing the immobility time in FST.	Quraishi et al. (2018)
quercetin 4′-O-glucoside	Mice	Swiss albino mice	CUMS	p.o	20 mg/kg	FST, OFT, SPT	Quercetin 4′-O-glucoside reduced the immobility time in FST and increased the number of line crossings in OFT, reversing the UCMS-induced decline in sucrose preference in SPT.	Singh et al. (2021)
Avicularin	Mice	C57BL/6 mice	CUMS	p.o	1.25, 2.5, 5.0 mg/kg	TST, FST, SPT	Avicularin reduced the immobility time of mice in TST and FST, increasing sucrose consumption in SPT.	Shen et al. (2019)

### 5.1 Isoquercetin

Isoquercetin is a quercetin derivative with glucose as the glycoside, which has been reported to have greater bioavailability. Morand et al. (2000) found that when quercetin was supplied to rats as isoquercetin, it was absorbed faster, tripling the plasma levels of quercetin metabolites. Furthermore, the antidepressant effect of isoquercetin is gradually being validated. For example, the depression-like behaviors of mice were alleviated after treatment with isoquercetin for 2 weeks, with the reduction of ACTH and CORT levels (Butterweck et al., 2004). Another study showed that isoquercetin, either in monotherapy or in combination with estrogen, could significantly improve oxidative stress, pro-inflammatory cytokines, and brain monoamine concentration in ovariectomized rats (Elnoury, 2019). Martínez-Hernández et al. (2021) found similar results. They showed that isoquercetin from American linden could exert antidepressant activity by resisting oxidative stress and antagonizing 5-HT1A receptors. Moreover, Dai et al. (2018) have shown that isoquercetin could inhibit oxidative stress by downregulating NF-E2-related factor 2 (Nrf2) and blocking the nicotinamide adenine dinucleotide phosphate NOX4/ROS/NF-κB pathway.

### 5.2 Hyperin

Hyperin, also known as quercetin-3-O-d-galactoside, is a major pharmacologically active component of *Hypericum perforatum* that has been demonstrated to have antidepressant effects (Patel et al., 2018). Previous experiments have shown that hyperin could attenuate depression-like behaviors in rats and mice models *via* the dopaminergic system (Haas et al., 2011; Zheng et al., 2012). Orzelska-Górka et al. (2019) found that the antidepressant effects of hyperin were partly mediated by the upregulation of the monoaminergic system and BDNF levels. Meanwhile, the effects of hyperin on reducing DA levels, regulating 5-HT receptors, and increasing BDNF expression have also been demonstrated by other studies (Aatz et al., 2021; Chen et al., 2021a; Jolodar et al., 2021). Additionally, hyperin has been reported with various pharmacological effects, such as anti-inflammatory, anti-oxidation, and reducing CORT levels. However, the correlation between those effects and depression improvement still needs further studies (Huang et al., 2015; Sevastre-Berghian et al., 2018). In contrast to quercetin, the regulatory effects of hyperin on the dopaminergic system have been confirmed (Kwon et al., 2019), that is, hyperin may achieve better efficacy in depression treatments.

### 5.3 Rutin

In recent years, several studies have demonstrated the antidepressant effects of rutin using different animal models (Yusha’u et al., 2017; Barauna et al., 2018; Daodee et al., 2019), especially by protecting hippocampal neurons (Parashar et al., 2017). Anjomshoa et al. (2020) found that rutin can also increase the diameter of the CA3 region in the hippocampus, whose mechanisms were different from quercetin and other derivatives. Moreover, rutin can improve depression by regulating the HPA axis (Quraishi et al., 2018). Graefe et al. (2001) have shown that, compared with other quercetin derivatives, rutin presented slow absorption and a high concentration peak in plasma. Another experiment demonstrated the slow onset of rutin by finding that the plasma levels of quercetin metabolites were low in rats 4 h after taking 20 mg of rutin powder (Morand et al., 2000), which is important for guiding the medication and formulating therapeutic doses of rutin.

### 5.4 Quercetin 4′-O-Glucoside

Quercetin 4′-O-Glucoside (QG) is the main quercetin of onions. The antidepressant effects of onion powder have been proved for a long time (Sakakibara et al., 2008). However, QG was not regarded as the main component responsible for the antidepressant effects of onions until 2021. Singh et al. (2021) found that QG could reduce depressive-like behaviors in CUMS mice, while MAO-A was inhibited and GSH and 5-HT levels were increased in the brain, indicating that the antidepressant effects of QG might be related to anti-oxidative stress and increased concentrations of monoamine in the brain. The antioxidant effects of QG have also been demonstrated in other studies (Murota et al., 2004; Chen et al., 2021b). Although QG has lower bioavailability compared with quercetin (Boonpawa et al., 2014), its content in plants is much higher, particularly in red onions where it can reach 13.2% (Arung et al., 2011). Therefore, the extraction of QG might cost less than quercetin (Wach et al., 2007). Additionally, Zheng et al. (2017) showed QG had higher antioxidant activity than quercetin through the sequential proton loss electron transfer (SPLET) mechanism, while its possible positive effects on humans remain to be elucidated.

### 5.5 Avicularin

Avicularin, also known as quercetin 3-O-*α*-l-arabinofuranoside, is a flavonoid and quercetin derivative (Guo et al., 2018). Compared with quercetin, avicularin has a stronger anti-inflammatory ability (Dung et al., 2016). The excessive secretion of cytokines in an inflammatory state will lead to depression. Therefore, the antidepressant potential of avicularin is worth studying. In an experiment performed by Shen et al. (2019), avicularin significantly alleviated depression-like behaviors in mice, similar to fluoxetine. They also found that the activation of the MEK/ERK/NF-κB signaling pathway in the hippocampus was inhibited, the levels of IL-1β, IL-6, and tumor necrosis factor-*α* (TNF-α) reduced, and neuronal apoptosis rate decreased after avicularin treatment. These findings demonstrated that avicularin could improve depression-like behaviors by inhibiting inflammation. However, studies on the antidepressant effects of avicularin focused only on its anti-inflammatory effects. Hence, it is necessary to study other possible mechanisms and compare the efficacy of avicularin with quercetin. Additionally, inflammation-induced depression is closely related to microglia, but no studies have focused on the relationship between avicularin and microglia, and have only used RAW 264.7 macrophages to demonstrate the role of avicularin in suppressing the inflammatory response (Van Anh Vo et al., 2012).

## 6 Discussion

Due to the outbreak of COVID-19, people around the world have suffered from the negative emotions caused by isolation, unemployment, and the sudden death of family members (Harper et al., 2020; Ustun, 2021). A recent study estimated that the COVID-19 pandemic led to additional 53.2 million cases of major depressive disorder globally, with an increase of total prevalence of 3,152.9 cases per 100,000 people (Santomauro et al., 2021). The unexpected stressors caused by the pandemic can also worsen the epidemiological status of depression. Nevertheless, depression treatments are still limited. For example, 46% of adults did not show improvement after taking antidepressants and 25–40% of patients who recover after treatment will experience depressive episodes again within 2 years, reaching 85% within 15 years (Cuijpers et al., 2020). Considering that a large proportion of patients taking antidepressants suffer from side effects and withdrawal reactions (Read and Williams, 2018), finding effective antidepressants with fewer side effects is urgent.

The latest Magnetic Resonance Imaging (MRI) results showed that the lesions in depression patients are localized in the frontal lobe, cingulate, hippocampus, and amygdala (Sindermann et al., 2021). Attributable to the discovery of the phenomenon of adult neurogenesis (Kempermann et al., 2018), the hippocampus has become the focus of studies as the seat of adult neurogenesis. Moreover, depression patients can present pathological changes such as hippocampal atrophy, reduction in the number of neurons and glial cells in the hippocampus. The degree of hippocampal atrophy is also positively correlated to the duration of depression symptoms (Bremner et al., 2000; Steffens et al., 2000). Patients with a small hippocampus have more difficulties achieving significant effects in antidepressant therapy (Hsieh et al., 2002). Therefore, two hypotheses for the pathogenesis of depression have been proposed: the neuroplasticity hypothesis and the regeneration hypothesis. The former refers to the atrophy of mature hippocampal neurons, and the latter refers to the reduction in the number of new neurons and neural precursor cells in the hippocampal dentate gyrus (Boku et al., 2018). Compared with the monoamine hypothesis, these two hypotheses have gained credibility among researchers by illuminating the mechanisms for the delayed action of antidepressants. However, the causal relationship between pathological changes in other brain regions and depression pathogenesis requires further study.

Recent studies have better explored the regeneration hypothesis. For example, Tunc-Ozcan et al. (2019) achieved antidepressant effects by stimulating new neurons without interfering with neurogenesis, while inhibiting the activity of newborn neurons can abrogate the effects of antidepressants, and no studies have demonstrated depression alleviation from the process of neurogenesis itself, suggesting the inhibition of the excitation of newborn neurons could replace the neurogenesis hypothesis as the mainstream view of depression pathogenesis. Nevertheless, these results do not affect the importance of neurogenesis since it is the basis for the excitation of new cells, and increasing neurogenesis remains important for relieving depression.

**FIGURE 3:**
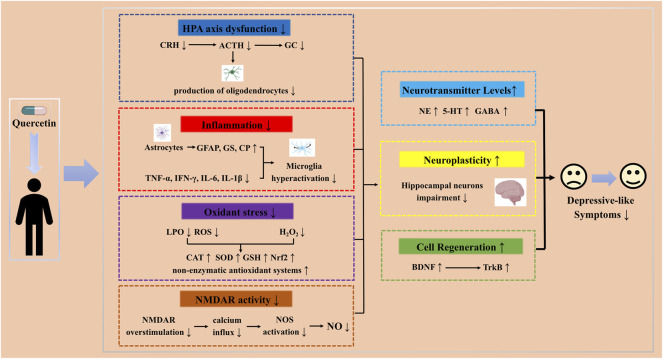
Schematic representation of different pathways and targets by quercetin as a potential therapeutic strategy in depression.

Antidepressants possibly exert their effects by protecting mature neurons and increasing neurogenesis in the hippocampus. The protection of mature neurons in the hippocampus is based on the improvement of HPA axis dysfunction, reducing inflammatory states and anti-oxidative stress, while the increase of hippocampal neurogenesis includes direct and indirect effects, similar to quercetin. As a flavonol with numerous biological activities, quercetin has different pharmacological effects such as anti-depression, anti-cancer, antioxidant, anti-fibrosis, and anti-inflammatory (Reyes-Farias and Carrasco-Pozo, 2019; Ou et al., 2020; Alshammari et al., 2021). Many studies have suggested the protection of mature neurons in the hippocampus, improvement of HPA axis dysfunction, anti-inflammatory, and anti-oxidative stress were involved in the antidepressant effects of quercetin (Sun et al., 2007; Bicca et al., 2021; El-Haroun et al., 2021; Han et al., 2021; Li et al., 2021; Sun et al., 2021), which also encompasses hippocampal neurogenesis promotion through direct and indirect effects (Chan et al., 2018; Rahvar et al., 2018; Wang et al., 2021a; Ma et al., 2021). Hence, quercetin has multiple targets and pathways compared to other antidepressants (Figure 3).

People who experience greater stress in their lives are at high risk for depression. Due to the lack of free time and money, most of them face the predicament that there are no effective measures to prevent depression. In this case, depression may be prevented using diets rich in quercetin and its derivatives. First, the antidepressant effects and their safety of quercetin and its derivatives as dietary supplements have been previously demonstrated (Andres et al., 2018; Babaei et al., 2018b). Additionally, foods with quercetin and its derivatives as main ingredients, such as onions and *Hypericum perforatum*, are readily available in daily life and have been demonstrated to have antidepressant effects (Di Pierro et al., 2018; RASTOGI et al., 2021). On the other hand, preventing depression through these foods can be more convenient and cost-effective. However, the intake required and how these foods should be consumed to prevent depression still need further study.

The biggest limitation of the current studies on the antidepressant effects of quercetin is the lack of clinical trials, which makes it difficult to determine the appropriate dose and duration. Considering other clinical studies on the treatment of depression with TCM, the combination of TCM and Western medicine generally leads to better efficacy than either of them alone. Hence, after fully considering the safety of quercetin and its glycoside derivatives, it would be suitable to explore whether they can have unexpected interactions with clinical first-line drugs in the future.

Quercetin and its derivatives generate quinone metabolites *via* tyrosinase *in vitro* (Awad et al., 2002), which cause false positives through metal chelation, oxidative cycling, and covalent binding (Baell and Nissink, 2018; Klopčič and Dolenc, 2018; Kato and Suga, 2018). Based on these results, quercetin and its derivatives were often mistaken for the active compounds of drugs in studies *in silico*, such as network pharmacology. However, it does not mean that quercetin does not have specific antidepressant effects, as the antidepressant activity of quercetin has been validated in numerous *in vivo* experiments (Zhang et al., 2020a; Şahin et al., 2020; Eduviere et al., 2021). This paradoxical phenomenon reminds us to be more cautious in future. First of all, as numerous studies *in vivo* have confirmed that quercetin could act on multiple targets in depression treatment (El-Haroun et al., 2021; Han et al., 2021; Li et al., 2021), the top priority of *in vitro* studies is not to prove whether quercetin has new targets in treating depression, but to reveal the mechanisms of the proved targets through more scientific models and methods. Besides, based on the difficulties to distinguish the non-specifically and therapeutically irrelevant effects (Baell, 2016), it is necessary to review the false positives and related mechanisms of quercetin, in order to guide researchers to select appropriate cells, culture mediums, control groups and evaluation methods. Furthermore, conclusions should be based on rigorous experimental validation, rather than results directly from *in silico* studies.

Moreover, the current studies still have inadequacies. Firstly, although the concentration of 5-HT is closely related to depression, 5-HT itself has no clinical application, since it can produce completely different pharmacological effects by binding to different receptors (Virk et al., 2016). In this case, demonstrating the regulation of 5-HT concentrations is not enough to explain the mechanisms behind antidepressants. On the other hand, studying the correlation between antidepressants and the expression of 5-HT receptors is of great significance. Although some studies have previously considered the relationship between quercetin and 5-HT receptors (Lee et al., 2005; Rotelli et al., 2009; Morales-Cano et al., 2014; Jakaria et al., 2019b), at least seven subtypes of 5-HT receptors are closely related to depression (Yohn et al., 2017), and most remain unexplored. Furthermore, Nugent et al. (2013) found that SSRIs can upregulate 5-HT levels in mice with 5-HT1A receptor deletion, but their depression-like behaviors did not improve. Hence, the relationship between quercetin and various 5-HT receptors still needs to be studied in depth. Secondly, SSRIs can increase the content of 5-HT in the synaptic cleft and are first-line drugs for depression treatment at present. However, studies investigating the relationship between quercetin and 5-HT have rarely used a control group treated with SSRIs. Therefore, the antidepressant effects of quercetin would be better illustrated using a positive control group treated with SSRIs. Moreover, there are numerous studies regarding the regulation of the HPA axis by quercetin, most of which were concentrated on the plasma content of CORT after quercetin treatment. Whether quercetin can reduce the overexpression of CORT receptors remains to be studied. Additionally, the mechanisms of BDNF regulation in the hippocampus are a hot research topic nowadays. Many single-stranded small non-coding ribonucleic acid (RNA) molecules, including miR-10b-5P (Wang et al., 2020), miR-202-3P (Xin et al., 2020), and miR-206-3P (Guan et al., 2021b) have been recently studied, as studies focused on the effects of other TCM on non-coding RNA molecules (Zhan et al., 2021). Research on the effects of quercetin on small non-coding RNA molecules has broad prospects. For quercetin glycoside derivatives, more exploration is required for their antidepressant effects and related mechanisms, as their multi-target and multi-pathway characteristics. Finally, from a practical point of view, the biotransformation rate should be the basis to study quercetin glycoside derivatives. Until now, studies have confirmed that isoquercetin has a superior biotransformation rate. Therefore, exploring its effects on neurons and promoting its clinical applicatiois of great interest.

Overall, the antidepressant effects of quercetin and its glycoside derivatives have been demonstrated by a large number of studies, and the related mechanisms have been continuously explored. After improving the studies regarding relevant mechanisms and safety, drugs based on quercetin and its glycoside derivatives can become the main components during depression treatment.
